# Clinical Phenotypes of Tumors Invading the Rectosigmoid Colon Affecting the Extent of Debulking Surgery and Survival in Advanced Ovarian Cancer

**DOI:** 10.3389/fonc.2021.673631

**Published:** 2021-04-22

**Authors:** Soo Jin Park, Jaehee Mun, Eun Ji Lee, Sunwoo Park, Sang Youn Kim, Whasun Lim, Gwonhwa Song, Jae-Weon Kim, Seungmee Lee, Hee Seung Kim

**Affiliations:** ^1^ Department of Obstetrics and Gynecology, Seoul National University College of Medicine, Seoul, South Korea; ^2^ Institute of Animal Molecular Biotechnology and Department of Biotechnology, College of Life Sciences and Biotechnology, Korea University, Seoul, South Korea; ^3^ Department of Radiology, Seoul National University College of Medicine, Seoul, South Korea; ^4^ Department of Food and Nutrition, Kookmin University, Seoul, South Korea; ^5^ Department of Obstetrics and Gynecology, Keimyung University Dongsan Hospital, Keimyung University School of Medicine, Daegu, South Korea

**Keywords:** phenotype, separability, rectosigmoid, outcomes, survival, ovarian cancer

## Abstract

We classified clinical phenotypes based on tumor separability from the rectosigmoid colon and then evaluated the effect of these clinical phenotypes on surgical outcomes and prognosis of advanced ovarian cancer. We collected data of patients with stage IIIB-IVB disease who either underwent visceral segmental serosectomy (VSS) or low anterior resection (LAR) during maximal debulking surgery. All patients were divided into the following, according to the resection types of tumors involving the rectosigmoid colon: the focal (tumor-involved length <18 cm) and separable (FS) group that received VSS, the focal and inseparable (FI) that received LAR, or the diffuse (tumor-involved length ≥18 cm) group (D) that also received LAR. A total of 83 patients were included in FS (n=44, 53%), FI (n=18, 21.7%), and D (n=24, 25.3%) groups. FS and D groups with more extensive tumors were related to wider extent of surgery and more tumor infiltration except for bowels, whereas FI and D groups with more invasive tumors were associated with wider extent of surgery, more tumor infiltration to bowels, longer operation time, more blood loss, more transfusion, longer hospitalization, and higher surgical complexity scores. Moreover, FS and FI groups showed better progression-free survival than D group, whereas FS group demonstrated better overall survival than FI and D groups. Clinical phenotypes based on tumor separability from the rectosigmoid colon may depend on tumor invasiveness and extensiveness in advanced ovarian cancer. Moreover, these clinical phenotypes may affect surgical outcomes and survival.

## Introduction

Maximal cytoreduction in advanced ovarian cancer is crucial (1, 2), however, removing tumors involving the rectosigmoid colon is burdensome for gynecologic oncologists during debulking surgery for advanced ovarian cancer. Only a few gynecologic oncologists themselves perform the removal of the tumor located in the rectosigmoid colon in clinical practice, while others commonly rely on colorectal surgeons in 80-90% of cases (3). However, tumors involving the rectosigmoid colon frequently appears in about 24-64% of patients undergoing debulking surgery in ovarian cancer (4, 5), and the incorporation of resecting tumors involving the rectosigmoid colon is still important to improve survival by complete resection of tumors (5–8).

For removing tumors involving the rectosigmoid colon, low anterior resection (LAR) is a popular method with no risk of leaving microscopic residual tumor. Nevertheless, anastomosis site leakage occurs in 2.3-6.8% of cases (9–11), and bowel diversion is required in 17-18% of patients undergoing LAR (10, 12). Also, it has been reported that 40% of patients who underwent LAR develop the low anterior resection syndrome, deteriorating quality of life (13, 14).

Even though stripping tumors involving the rectosigmoid colon can be an alternative to LAR because the rectosigmoid colon can be preserved, and it is more feasible for gynecologic oncologists to conduct stripping tumors, controversy about its safety has been raised in several studies. Such studies argue that the removal of tumors without resecting the rectosigmoid colon can leave microscopic tumors, leading to a decreased survival rate (15, 16). On the contrary, recent studies support that stripping tumors involving the rectosigmoid colon may not worsen the prognosis of advanced ovarian cancer when compared to LAR (17–19).

Thus, we initially designed this study to evaluate the safety and feasibility of visceral segmental serosectomy (VSS), one of the methods for stripping tumors and compared it to LAR in advanced ovarian cancer. However, we found that LAR was inevitably required in some cases despite the attempt to perform VSS because the rectosigmoid colon could not be preserved due to tumor invasiveness and extensiveness, which was associated with the prognosis of advanced ovarian cancer (20).

Finally, we hypothesized that the possibility of exfoliating tumors while preserving the rectosigmoid colon might depend on clinical phenotypes based on tumor separability rather than the skill of surgeons, and these clinical phenotypes depending on tumor invasiveness and extensiveness might be associated with surgical outcomes and the prognosis of advanced ovarian cancer. Therefore, this study aims to show the effect of these clinical phenotypes according to tumor separability from the rectosigmoid colon on surgical outcomes and survival in patients with advanced ovarian cancer.

## Materials and Methods

### Patient Selection

We identified patients diagnosed with epithelial ovarian cancer from August 2014 to June 2020 at Seoul National University Hospital. Among those, we included only patients who; had International Federation of Obstetrics and Gynecology (FIGO) stage IIIB and IVB disease; underwent maximal debulking surgery for primary disease by one experienced gynecologic oncologist (HSK) only to control the quality of the data; was assessed for tumor separability in the rectosigmoid colon during debulking surgery with the attempt of preserving it. Seoul National University Hospital Institutional Review Board (IRB) approved this study (H-1707-079-869), and the patient consent was waived due to a retrospective design.

### Surgical Procedure

**Figure 1:**
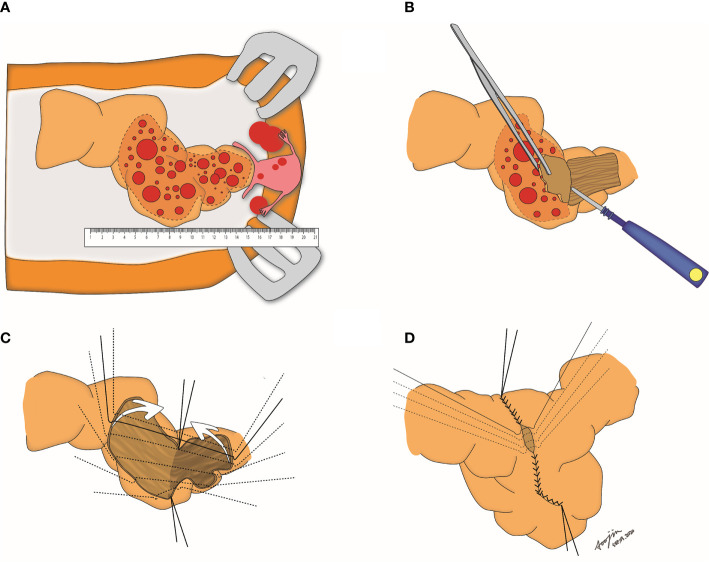
Step-by-step procedures of visceral serosal segmentectomy. **(A)** Gross evaluation of the length of tumors involving the rectosigmoid colon; **(B)** Stripping of the tumors by electrocauterization; **(C)** Both right and left anterolateral edges are tagged, and the stripped surface of the bowel is folded; **(D)** Edges of the stripped area are closed with 3-0 vicryl sutures.

**Figure 2:**
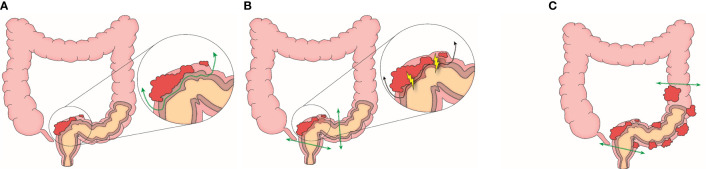
Clinical phenotypes by tumor separability **(A)** Focal and separable type **(B)** Focal and inseparable type **(C)** Diffuse type.

All patients underwent debulking surgery for optimal cytoreduction according to the procedure described in the previous study (21). During the surgery, the operator (HSK) evaluated the length of tumor involvement in the rectosigmoid colon. When the tumor-involved length was less than 18cm, we initially attempted VSS to leave the rectosigmoid colon intact according to the surgical procedure reported previously (**Figure 1**) (20). When the tumor could be separated easily without inflicting mucosal defect by VSS, it was defined as the focal and separable (FS) type. On the other hand, we converted from VSS to LAR when a mucosal defect occurred during the procedure, defined as the focal and inseparable (FI) type. Lastly, we performed LAR when the tumor-involved length exceeded 18 cm due to the risk of leakage at the serosa repair site by excessive tension, which was defined as the diffuse (D) type (**Figure 2**). The tumor separability depending on tumor invasiveness and extensiveness was verified by two independent gynecologic oncologists (SJP and EJL), and the third gynecologic oncologist (JM) resolved disagreement between the two verifiers during debulking surgery. Three gynecologic oncologists performed verification using surgical records, pathology reports, and videos and photographs of the surgical fields uploaded to electronic medical records.

### Data Collection

Depending on the resection types of tumors involving the rectosigmoid colon, we divided all patients into FS, FI, and D groups. Then, we collected data of the three groups as follows: age, FIGO stage, histologic types, treatment types, use of bevacizumab, duration of follow-up, surgical extent, tumor infiltration, operative outcomes, progression-free survival (PFS), and overall survival (OS). We calculated the surgical complexity score (SCS) to evaluate the complexity and the extent of debulking surgery (22) and assessed surgical complications by using the Memorial Sloan Kettering Cancer Center (MSKCC) surgical secondary event grading classifications (23). Tumor response was assessed by the Response Evaluation Criteria In Solid Tumors (RECIST) criteria (version 1.1) (24). FS was defined as the time interval from the treatment start date to the disease recurrence or last follow-up date, while OS was defined as the time interval from the treatment start date to the cancer-related death or last follow-up date. The recurrence pattern was classified into the tumor resection site on the rectosigmoid colon, the pelvic cavity, the abdominal cavity, and distant metastasis.

### Statistical Analysis

The Chi-squared test was done for categorical variables, and the Kruskal-Wallis test was done for continuous variables. Survival outcome was estimated by the Kaplan-Meier method with the log-rank and Breslow tests, and factors related to survival were identified by the Cox proportional hazard regression method using hazard ratio (HR) and 95% confidence interval (CI). We performed all statistical analysis using SPSS software version 22.0 (SPSS Inc., Chicago, IL, USA).

## Results

### Baseline Characteristics

A total of 83 patients underwent maximal debulking surgery, including the resection of the tumors involving the rectosigmoid colon for advanced ovarian cancer. Among them, 44 (53%), 18 (21.7%), and 24 (25.3%) patients were assigned to FS, FI, and D groups, respectively. There were no differences in age, FIGO stage, histology, treatment types, use of bevacizumab, and follow-up duration among the three groups (**Table 1**).

**Table 1:** Clinicopathologic characteristics.

	FS group(n=44, %)	FI group(n=18, %)	D group(n=21, %)	*P* value
Age (median, range, years)	57 (32, 76)	61 (31, 89)	60 (42, 79)	0.821
FIGO stage				0.126
III	17 (38.6)	12 (66.7)	9 (42.9)	
IV	27 (61.4)	6 (33.3)	12 (57.1)	
Histology				0.289
Serous	39 (88.6)	14 (77.8)	21 (100)	
Endometrioid	0	1 (5.6)	0	
Clear cell	3 (6.8)	3 (16.7)	0	
Mixed	2 (4.6)	0	0	
Treatment				0.239
Neoadjuvant chemotherapy	25 (56.8)	13 (72.2)	16 (76.2)	
Primary debulking surgery	19 (43.2)	5 (27.8)	5 (23.8)	
Use of bevacizumab				0.674
Yes	5 (11.4)	1 (5.6)	3 (14.3)	
No	39 (88.6)	17 (94.4)	18 (85.7)	
Duration of follow-up (median, range, mons)	45.1 (8.3-70.3)	64.0 (6.7-128.7)	38.7 (9.4-80.4)	0.139

D, diffuse; FI, focal and inseparable; FIGO, International Federation of Gynecology and Obstetrics; FS, focal and separable.

### Surgical Extent and Tumor Infiltration

**Table 2** reveals the surgical extent and tumor infiltration in the resected areas. Splenectomy, cholecystectomy, portal triad stripping, left diaphragm peritonectomy, and right and left paracolic peritonectomy were performed more commonly in D group than in FI group. However, there was no difference in small and large bowel resection between D and FI groups. On the other hand, FS group underwent right and left paracolic peritonectomy more commonly than FI group and received small bowel resection and prophylactic ileostomy more frequently than D group.

**Table 2:** Surgical extent and tumor infiltration.

Surgical Extent	FS group (n=44, %)	FI group(n=18, %)	D group (n=21, %)	p-value	Tumor infiltration	FS group (n=44, %)	FI group (n=18, %)	D group (n=21, %)	p-value
Hysterectomy	44 (100)	18 (100)	21 (100)	–	Uterus	29 (65.9)	13 (72.2)	18 (85.7)	0.249
BSO	44 (100)	18 (100)	21 (100)	–	Adnexa	43 (97.7)	18 (100)	20 (95.2)	0.624
Lymphadenectomy	44 (100)	18 (100)	21 (100)	–	Lymph node	28 (63.6)	10 (55.6)	13 (61.9)	0.838
Omentectomy	44 (100)	18 (100)	21 (100)	–	Omentum	26 (59.1)	10 (55.6)	17 (81)	0.163
Hepatobiliary resection					Hepatobiliary resection				
Splenectomy	27 (61.4)a,b	7 (38.9)a	17 (81.0)b	0.027	Spleen	18 (40.9)	5 (27.8)	12 (57.1)	0.175
Distal pancreatectomy	12 (27.3)	1 (5.6)	6 (28.6)	0.14	Distal pancreas	5 (11.4)	0	2 (9.5)	0.336
Hepatectomy	1 (2.3)	1 (5.6)	0	0.527	Liver (hepatectomy)	1 (2.3)	1 (5.6)	0	0.527
Wedge resection of liver	11 (25.0)	2 (11.1)	7 (33.3)	0.265	Liver (wedge resection)	9 (20.5)o,p	1 (5.6)o	7 (33.3)p	0.101
Cholecystectomy	14 (31.8)c,d	2 (11.1)c	10 (47.6)d	0.049	Gall bladder	8 (18.2)	0	4 (19)	0.143
Portal triad stripping	11 (25)e,f	1 (5.6)e	9 (42.9)f	0.028	Portal triad	9 (20.5)q	0r	4 (19)q,r	0.117
Adrenalectomy	5 (11.4)	1 (5.6)	4 (19.0)	0.426	Adrenal gland	2 (4.5)	1 (5.6)	1 (4.8)	0.986
Cardiophrenic LND	15 (34.1)	3 (16.7)	6 (28.6)	0.389	Cardiophrenic LN	12 (27.3)	2 (11.1)	2 (9.5)	0.145
Peritonectomy					Peritonectomy				
Rt diaphragm peritonectomy	30 (68.2)	10 (55.6)	16 (76.2)	0.386	Rt diaphragm peritoneum	27 (61.4)	10 (55.6)	16 (76.2)	0.361
Lt diaphragm peritonectomy	20 (45.5)g,h	4 (22.2)g	13 (61.9)h	0.045	Lt diaphragm peritoneum	19 (43.2)s,t	4 (22.2)s	13 (61.9)t	0.045
Rt paracolic peritonectomy	30 (68.2)i	5 (27.8)	14 (66.7)i	0.01	Rt paracolic peritoneum	26 (59.1)u	4 (22.2)v	10 (47.6)u,v	0.031
Lt paracolic peritonectomy	26 (59.1)j	4 (22.2)	13 (61.9)j	0.017	Lt paracolic peritoneum	23 (52.3)w	4 (22.2)	12 (57.1)w	0.055
Rt pelvic peritonectomy	30 (68.2)	9 (50)	13 (61.9)	0.404	Rt pelvic peritoneum	26 (59.1)	7 (38.9)	11 (52.4)	0.35
Lt pelvic peritonectomy	28 (63.6)	8 (44.4)	12 (57.1)	0.38	Lt pelvic peritoneum	23 (52.3)	7 (38.9)	11 (52.4)	0.602
Bladder peritonectomy	30 (68.2)	14(77.8)	17 (81.0)	0.495	Bladder peritoneum	28 (63.6)	12 (66.7)	16 (76.2)	0.598
Bowel surgery					Bowel surgery				
Small bowel R&A	4 (9.1)k	4 (22.2)k,l	7 (33.3)l	0.052	Small bowel segment	4 (9.1)x	2 (11.1)x,y	6 (28.6)y	0.102
Large bowel R&A	10 (22.7)	4 (22.2)	8 (38.1)	0.379	Large bowel segment	6 (13.6)z	3 (16.7)z,aa	8 (38.1)aa	0.066
Appendectomy	27 (61.4)	9 (50)	16 (76.2)	0.234	Appendix	17 (38.6)bb,cc	4 (22.2)bb	13 (61.9)cc	0.038
Prophylactic ileostomy	1 (2.3)m	2 (11.1)m,n	4 (19.0)n	0.067					

BSO, bilateral salpingo-oophorectomy; D, diffuse; FI, focal and inseparable; FS, focal and separable; LND, lymph node dissection; LN, lymph node; R&A, resection and anastomosis.

There is no significant difference between the two groups with the same symbols.

In pathologic examination, D group showed tumor infiltration more commonly in the left diaphragm, left paracolic peritoneum, and appendix than FI group. However, there was no difference in tumor infiltration in the small and large bowel segments between the two groups. On the other hand, FS group showed tumor infiltration more frequently in the right and left paracolic peritoneum than FI group and demonstrated it less common in the small and large bowel segments than D group.

### Operative Outcomes

**Table 3** depicts the comparison of operative outcomes between the three groups. First, FI and D groups showed the tendency of longer operation time, more blood loss, more transfusion, and longer hospitalization than FS group. The SCS was highest in D group, followed by FI and FS groups. However, there were no differences in the residual tumor size and the time interval from surgery to adjuvant chemotherapy among the three groups.

**Table 3:** Operative outcomes.

Outcomes	FS group (n=44, %)	FI group (n=18, %)	D group (n=21, %)	p-value
Operation time (median, range, minutes)	300 (170, 761)a	343 (215, 630)a,b	440 (200, 785)b	0.008
Estimated blood loss (median, range, ml)	1450 (300, 5000)c	1615 (350, 6900)c	2800 (350, 9000)	0.008
Transfusion (median, range)	3 (0, 11)d	4 (0, 15)d,e	6 (1, 16)e	0.007
Hospitalization (median, range, days)	13 (8, 36)f	13.5 (9, 46)f,g	17 (11, 38)g	0.019
Surgical Complexity Score (median, range)	10 (4, 14)	10.5 (7, 17)	14 (7, 18)	<0.001
Size of residual tumor				
No gross residual tumor	35 (79.5)	15 (83.3)	14 (66.7)	0.398
<0.5cm	42 (95.5)	16 (88.9)	17 (81.0)	0.175
<1cm	44 (100)	17 (94.4)	19 (90.5)	0.139
Time interval from surgery to adjuvant chemotherapy (median, range, days)	27 (10, 56)	25 (11, 70)	27 (12, 51)	0.429
Surgical complication				
Gastrointestinal				0.298
Grade 1-2	2 (4.5)	1 (5.6)	0 (0)	
Grade 3-4	0 (0)	1 (5.6)	2 (9.5)	
Infection				0.023
Grade 1-2	4 (9.1)	1 (5.6)	2 (9.6)	
Grade 3-4	0 (0)	3 (16.7)	0 (0)	
Thromboembolic				0.599
Grade 1-2	2 (4.5)	0 (0)	0 (0)	
Grade 3-4	1 (2.3)	0 (0)	0 (0)	
Complication at the tumor resection site of the rectosigmoid colon (n, %)				
Leakage	0 (0)	1 (5.6)	2 (9.5)	0.139
Bleeding	0 (0)h	2 (11.1)i	1 (4.8)h,i	0.099

D, diffuse; FI, focal and inseparable; FS, focal and separable.

There is no significant difference between the two groups with the same symbols.

Regarding surgical complications, grade 3-4 infection was more common in the FI group than in FS and D groups, whereas there were no differences in gastrointestinal and thromboembolic complications among the three groups. Moreover, there were no differences in leakage and bleeding at the tumor resection site of the rectosigmoid colon among the three groups.

### Survival

**Figure 3:**
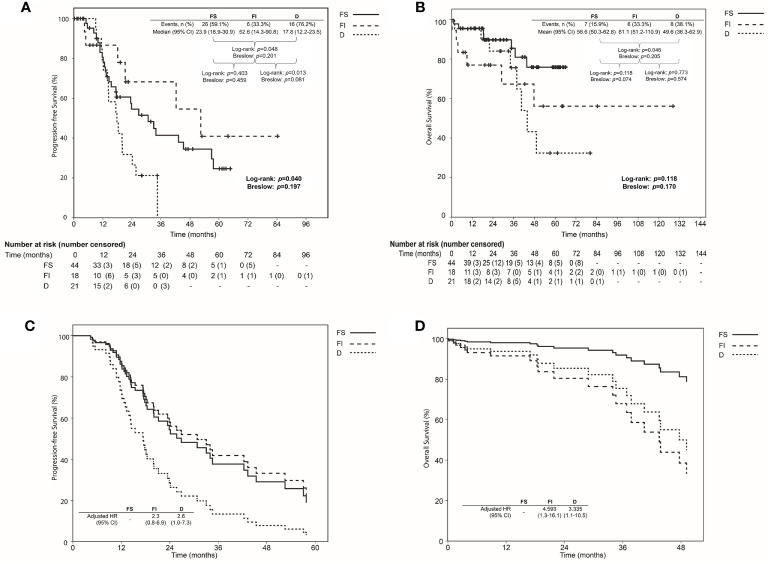
Comparison of survival using Kaplan-Meier method with log-rank and Breslow tests among the focal and separable (FS), focal and inseparable (FI), and diffuse groups **(D)** based on the resection types of tumor involving the rectosigmoid colon: **(A)** Progression-free survival; **(B)** Overall survival; Comparison of the survival proportion by Cox proportional hazards regression analysis among the focal and separable (FS), focal and inseparable (FI), and diffuse groups **(D)** based on the resection types of tumor involving the rectosigmoid colon: **(C)** Progression-free survival; **(D)** Overall survival.

Median values of PFS were 23.9, 52.6, and 17.8 months, and mean values of OS were 56.6, 81.1, and 49.6 months in FS, FI, and D groups, respectively (**Figure 3**). The Kaplan-Meier curve for PFS showed significant differences between FS and FI groups (p=0.048) and between FI and D groups (p=0.013). Although the Kaplan-Meier curve for OS of the three groups did not show significant differences, a significant difference was shown between FS and D groups (p=0.046), and the marginal difference was shown between FS and FI groups (p=0.074). When we evaluated hazards of disease progression and cancer-related death, D group showed a higher hazard of disease progression than FS and FI groups, while FI and D groups demonstrated a higher hazard of cancer-related death than FS group (**Figure 3**).

**Table 4** presents the factors affecting PFS and OS. Primary debulking surgery, no gross residual tumor, and D type affected PFS (adjusted HRs, 0.44, 0.52, and 2.06; 95% CIs, 0.23-0.85, 0.25-1.08 and 1.01-4.20). Furthermore, stage III disease, no gross residual tumor, and FI and D types affected OS (adjusted HRs, 0.25. 0.24, 4.59 and 3.34; 95% CIs, 0.08-0.81, 0.08-0.70, 1.31-16.08, and 1.06-10.54).

**Table 4:** Factors affecting progression-free survival and overall survival.

	Univariate		Multivariate	
	**HR (95% CI)**	**p-value**	**Adjusted HR (95% CI)**	**p-value**

Progression-free survival				
Stage III disease	0.53 (0.30-0.95)	0.03	0.75 (0.39-1.43)	0.38
Primary debulking surgery	0.43 (0.23-0.78)	0.01	0.44 (0.23-0.85)	0.01
Serous type	0.64 (0.23-1.811)	0.40	1.00 (0.34-2.93)	0.99
Age ≤57 years	0.83 (0.47-1.48)	0.54	0.89 (0.49-1.64)	0.71
No gross residual tumor	0.34 (0.18-0.65)	<0.001	0.52 (0.25-1.08)	0.08
Surgery on the rectosigmoid colon				
FI type	0.67 (0.28-1.63)	0.38	0.89 (0.34-2.36)	.82
D type	1.96 (1.02-3.76)	0.04	2.06 (1.01-4.20)	.04
Overall survival				
Stage III disease	0.31 (0.12-0.80)	0.02	0.25 (0.08-0.81)	0.02
Primary debulking surgery	0.52 (0.21-1.28)	0.16	0.65 (0.22-1.89)	0.43
Serous type	0.53 (0.18-1.59)	0.26	0.29 (0.08-1.04)	0.06
Age ≤57 years	0.59 (0.25-1.41)	0.23	0.47 (0.18-1.23)	0.12
No gross residual tumor	0.18 (0.08-0.44)	<0.001	0.24 (0.08-0.70)	0.01
Surgery on the rectosigmoid colon				
FI type	2.32 (0.78-6.92)	0.13	4.59 (1.31-16.08)	0.02
D type	2.65 (0.96-7.33)	0.06	3.34 (1.06-10.54)	0.04

D, diffuse; FI, focal and inseparable; HR, hazard ratio; CI, confidence interval.

### Recurrence Pattern

Among 48 patients (57.8%) with disease recurrence, 26 (59.1%), 6 (33.3%), and 16 (76.2%) were included in FS, FI, and D groups. There was no difference in the recurrence pattern among the three groups, and disease recurrence at the rectosigmoid colon tumor resection site was not observed (**Table 5**).

**Table 5:** Recurrence pattern.

Recurrent sites	FS group (n=26, %)	FI group (n=6, %)	D group (n=16, %)	P value
Tumor resection site of the rectosigmoid colon (n, %)	0 (0)	0 (0)	0 (0)	0.088
Pelvis other than the rectosigmoid colon (n, %)	2 (7.7)	1 (16.7)	1 (6.3)	
Intra-abdominal above the pelvis (n, %)	7 (26.9)	4 (66.7)	10 (62.5)	
Distant metastasis (n, %)	17 (65.4)	1 (16.7)	5 (31.3)	

D, diffuse; FI, focal and inseparable; FS, focal and separable.

## Discussions

This study demonstrates three clinical phenotypes of advanced ovarian cancer, showing differences in operative outcome, surgical extent, and survival. **Table 6** summarizes the main findings of three clinical phenotypes based on resection types of tumors involving the rectosigmoid colon, which may affect surgical extent, tumor infiltration, operative outcomes, and even the prognosis of advanced ovarian cancer in this study.

**Table 6:** Clinical phenotypes based on the resection types of tumors involving the rectosigmoid colon.

Characteristics	FS group	FI group	D group
Clinical phenotypes	Less invasive but more extensive	More invasive but less extensive	More invasive and more extenstive
Surgical extent			
Bowels	+	++	++
Others	++	+	++
Tumor infiltration			
Bowels	+	++	++
Others	++	+	++
Operative outcomes			
Operation time	+	+ or ++	++
Estimated blood loss	+	+	++
Transfusion	+	+ or ++	++
Hospitalization	+	+ or ++	++
Surgical complexity	+	++	+++
Risk of disease progression	Lower	Lower	Higher
Risk of cancer-related death	Lower	Higher	Higher

D, diffuse; FI, focal and inseparable; FS, focal and separable.

Like this study, the association between the invasion depth of tumors involving the rectosigmoid colon and the prognosis of advanced ovarian cancer has been discussed in previous studies (25–27). Although a previous study has shown no difference in survival between the invasion to the serosal or subserosal layer and that to the mucosal or muscularis mucosal layer in patients with advanced ovarian cancer who underwent LAR with no residual tumor (25), other studies have demonstrated that tumor invasion to no deeper than the serosal layer was related with a better prognosis (26, 27).

However, our study focused on tumor separability, not the depth of tumor invasion in the rectosigmoid colon, because tumor separability may be more critical in determining whether the rectosigmoid colon can be preserved. Even though the invasion of tumors to the longitudinal or circular muscle layer was often observed, we could preserve the rectosigmoid colon without opening the mucosal layer in FS group, whereas it could not be preserved in FI group (20). This means that separable tumors were mainly distributed in FS group, where gynecologic oncologists could have less burden and try to preserve the rectosigmoid colon during debulking surgery for advanced ovarian cancer. On the other hand, tumors in FI and D groups were more invasive than those in FS group, which needed LAR instead of VSS. Moreover, FI group was considered to be in the initial stage, whereas D group was in the progressive stage in terms of tumor extensiveness despite the similar characteristics of tumor invasiveness.

Among these clinical phenotypes, FS and D groups with more extensive tumors were related to wider extent of surgery and more tumor infiltration except bowels in this study. Only one study compared the surgical extent between patients who underwent conservative surgery on the rectosigmoid colon and those treated with LAR (19), which showed no difference in the surgical extent between the two groups, suggesting that the resection types of tumors involving the rectosigmoid colon may not be related with the surgical extent. However, more relevant studies are needed because the rate of upper abdominal surgery, reflecting a large volume of tumors, was low in the previously mentioned study when compared with our study (16 vs. 68.2%).

On the other hand, FI and D groups with more invasive tumors were associated with wider extent of surgery and more tumor infiltration to bowels. Moreover, the two groups were related to longer operation time, more blood loss, more transfusion, and longer hospitalization, reflecting that debulking surgery was more complex than FS group. Previous studies can support these findings where patients treated with LAR showed longer operation time, more blood loss, and longer hospitalization than those treated with conservative surgery (18, 19).

There were no differences in leakage and bleeding among the three groups in terms of complications at the rectosigmoid colon tumor resection site. Previous reports showed that the increasing number of bowel resections might be associated with higher morbidity (28, 29), suggesting that a higher risk of complications such as leakage may increase after LAR. Our study also found that three patients (7.7%) resulted in leakage and bleeding after LAR, whereas there was no leakage and bleeding after VSS. This means that VSS can be effective and safe for resecting tumors and preserving the rectosigmoid colon in patients with advanced ovarian cancer.

In terms of survival, we found that tumor invasiveness rather than tumor extensiveness was related to PFS and OS in patients. In a previous study, stripping tumors was associated with worse PFS than LAR, suggesting that the remnant microscopic disease by partial resection of tumors involving the rectosigmoid colon can act as a disease recurrence point (15). However, no gross residual tumor instead of R0 resection is the goal of optimal debulking surgery for advanced ovarian cancer (3, 30), and there was no difference in survival between conservative surgery and LAR based on this goal in other studies (17–19). Another previous study revealed the association between metastasis of mesenteric lymph node and rectosigmoid colon resection, but survival was not affected (31). Also, our study suggests that less invasive tumors involving the rectosigmoid colon may be one of the most important factors related to better PFS and OS in patients with advanced ovarian cancer, and less extensive tumors may be associated with better PFS in patients with more invasive tumors involving the rectosigmoid colon.

These clinical phenotypes based on resection types of tumors involving the rectosigmoid colon can have different gene expressions. Previous studies have suggested the differences of 21 gene expressions in ovarian cancer presenting bowel metastasis (32) and molecular features reflecting clinical phenotypes such as complete cytoreduction and survival (33). The results from our study also suggest that clinical phenotypes with regard to resection types of tumors involving the rectosigmoid colon are worthy of being investigated up to the molecular levels of ovarian cancer. The added value of our results on current literature is that the novel concept of clinical phenotypes dividing advanced ovarian cancer and the patient groups based on clinical phenotypes showed different surgical outcomes and prognoses in terms of PFS and OS. Therefore, further studies based on finding molecular biomarkers associated with the clinical phenotype is warranted.

In conclusion, clinical phenotypes based on the invasiveness and extensiveness of tumors in the rectosigmoid colon may affect the surgical extent, tumor infiltration, operative outcomes, and prognosis of advanced ovarian cancer. The strength of our study is that we presented a novel concept of clinicHal phenotyping of advanced ovarian cancer, verified by independent gynecologic specialists. Also, we showed the clinical significance of the phenotypes with operative and prognostic outcomes within patients who underwent consistent surgical techniques. When we consider the limitations of this study, such as a small number of patients and a retrospective design, further studies are needed to prove the significance of these clinical phenotypes and to find relevant biomarkers reflecting those.

## Data Availability Statement

The raw data supporting the conclusions of this article will be made available by the authors, without undue reservation.

## Ethics Statement

The studies involving human participants were reviewed and approved by Seoul National University Hospital Institutional Review Board (IRB). The ethics committee waived the requirement of written informed consent for participation.

## Author Contributions

SJP: methodology, validation, formal analysis, investigation, data curation, writing-original draft, and visualization. JM: validation, investigation, and writing-review and editing. EL: validation, investigation, and writing-review and editing. SP: validation, investigation, and writing-review and editing. SK: methodology, validation, investigation, and supervision. WL: validation, investigation, and writing-review and editing. GS: methodology, validation, investigation, and supervision. J-WK: methodology, validation, investigation, and supervision SL: methodology, validation, investigation, resources, data curation, and writing-review and editing. HK: conceptualization, methodology, formal analysis, resources, writing-review and editing, supervision, project administration, and funding acquisition. All authors contributed to the article and approved the submitted version.

## Funding

This study was supported by grants from Seoul National University (No. 800-20190437 and 800-20200309) and from Seoul National University Hospital (No. 0620173960).

## Conflict of Interest

The authors declare that the research was conducted in the absence of any commercial or financial relationships that could be construed as a potential conflict of interest.
